# Making recombinant proteins in filamentous fungi- are we expecting too much?

**DOI:** 10.3389/fmicb.2014.00075

**Published:** 2014-02-27

**Authors:** Helena Nevalainen, Robyn Peterson

**Affiliations:** Biomolecular Frontiers Research Centre, Department of Chemistry and Biomolecular Sciences, Macquarie University, SydneyNSW, Australia

**Keywords:** filamentous fungi, recombinant proteins, expression, secretion, *Trichoderma reesei*

## Abstract

Hosts used for the production of recombinant proteins are typically high-protein secreting mutant strains that have been selected for a specific purpose, such as efficient production of cellulose-degrading enzymes. Somewhat surprisingly, sequencing of the genomes of a series of mutant strains of the cellulolytic *Trichoderma reesei*, widely used as an expression host for recombinant gene products, has shed very little light on the nature of changes that boost high-level protein secretion. While it is generally agreed and shown that protein secretion in filamentous fungi occurs mainly through the hyphal tip, there is growing evidence that secretion of proteins also takes place in sub-apical regions. Attempts to increase correct folding and thereby the yields of heterologous proteins in fungal hosts by co-expression of cellular chaperones and foldases have resulted in variable success; underlying reasons have been explored mainly at the transcriptional level. The observed physiological changes in fungal strains experiencing increasing stress through protein overexpression under strong gene promoters also reflect the challenge the host organisms are experiencing. It is evident, that as with other eukaryotes, fungal endoplasmic reticulum is a highly dynamic structure. Considering the above, there is an emerging body of work exploring the use of weaker expression promoters to avoid undue stress. Filamentous fungi have been hailed as candidates for the production of pharmaceutically relevant proteins for therapeutic use. One of the biggest challenges in terms of fungally produced heterologous gene products is their mode of glycosylation; fungi lack the functionally important terminal sialylation of the glycans that occurs in mammalian cells. Finally, exploration of the metabolic pathways and fluxes together with the development of sophisticated fermentation protocols may result in new strategies to produce recombinant proteins in filamentous fungi.

## INTRODUCTION

As scavengers of recalcitrant polymers in nature, filamentous fungi such as the cellulolytic *Trichoderma reesei* are exceptionally good secretors of proteins outside the growing hyphae. Over the years, this property has been improved to the extent that current industrial production strains are well capable of secreting of the order of 100 g/L homologous proteins into the cultivation medium under optimized fermentation conditions (Cherry and Fidantsef, 2003). As these levels are far better than with any other organism, filamentous fungi hold the promise for an ultimate production host for recombinant proteins on an industrial scale. Toward this end, current research is carried out into cellular mechanisms for internal protein quality control, secretion stress, functional genomics relating to protein expression and secretion, post-translational protein modification, application of alternative expression promoters, identification of specific transcription factors and linking the fungal physiology to productivity (reviewed in Punt et al., 2002; Meyer, 2008; Lubertozzi and Keasling, 2009; Sharma et al., 2009; Fleissner and Dersch, 2010; Schuster and Schmoll, 2010; Ward, 2012). Molecular approaches such as optimizing the codon usage and expressing foreign proteins as a fusion to homologous highly secreted proteins, have become a routine practice in fungal laboratories (Conesa et al., 2001; Nevalainen et al., 2004).

Apart from the yield, it is equally important that a given heterologous protein is produced in an active/functional form. Proteins secreted from filamentous fungi are modified in the secretory pathway by folding, proteolytic processing and addition of glycans as the main modifications. From the point of view of making functional recombinant proteins of mammalian origin in filamentous fungi, the most crucial modification is perhaps glycosylation as it may affect the functionality, serum half-life and immunogenicity of a given protein. The main mode of glycosylation in filamentous fungi is of the high-mannose type and the added sugars do not feature the functionally important terminal sialylation of the glycans that occurs in mammalian cells (Brooks, 2004; Deshpande et al., 2008; De Pourcq et al., 2010). Potential developments in this field will be discussed below.

Experimental evidence suggests that a considerable number foreign proteins expressed in filamentous fungi is lost or stuck in the secretory pathway because of incorrect processing, modification or misfolding that results in their elimination by cellular quality control mechanisms (Archer and Peberdy, 1997; Gouka et al., 1997). Genetic, transcriptomic and proteomic studies into these mechanisms have revealed several genes and regulatory circuits active in the process (Chapman et al., 1998; Pakula et al., 2003; Al-Sheikh et al., 2004). This knowledge inspired a series of papers involving overexpression of genes encoding cellular foldases and chaperones in *Aspergillus niger*, co-expressed with a heterologous gene of interest (see Nevalainen et al., 2004). The results varied from “no effect” (e.g., *prpA *with calf chymosin; Wang and Ward, 2000) to *pdiA *with plant thaumatin resulting in fivefold yield improvement (Moralejo et al., 2001). The number of published examples is too low to make any wider conclusions but suggests that the yields of heterologous fungal proteins have a better chance for improvement in a filamentous fungal host than proteins of mammalian origin; the same holds true with heterologous proteins that have been produced without a chaperon-boost.

A typical lifecycle for an industrially applied filamentous fungus includes vegetative hyphae and asexual conidia that germinate forming new hyphae, or more rarely, production of sexual spores that undergo meiosis. Proteins are mainly secreted through the growing hyphal tip, making growth and protein secretion intimately linked and thus difficult to study separately (Wessels, 1993). The transport machinery in actively growing hyphae is required to function efficiently to ensure that cell wall material needed for growth is available in hyphal tips, a function that also links to secretion of extracellular proteins. There are many examples of growth rate-associated production of secreted proteins in filamentous fungi, e.g., production of α-amylase by *A. oryzae* (Spohr et al., 1998; Carlsen and Nielsen, 2001) and glucoamylase production by *A. niger *(Schrickx et al., 1993; Withers et al., 1998; Pedersen et al., 2000). Low growth rates in chemostat cultures of *T. reesei* generally correlated with an increase in the production of the extracellular proteins. However, protein production decreased at very low growth rates when a rather large part of the carbon source consumed was probably used for maintenance requirements of the cell (Pakula et al., 2005). There is also evidence that starvation will induce enzyme secretion as a quest for the fungus to find food (Gong et al., 1979; Mach et al., 1999). These findings underline the importance of adjusting the cultivation parameters to create physiological conditions that support protein production.

The transforming DNA is typically integrated as part of the fungal genome as alternation between the hyphal growth and formation of uni- or multinuclear conidia makes it hard to maintain a population of autonomously replicating plasmids, for example, to boost the gene copy numbers and thereby product yields. Having said this, some fungi do possess autonomous replication sequence (ARS) elements (e.g., *Fusarium oxysporum*, Powell and Kistler, 1990; *A. nidulans, *Gems et al., 1991; Aleksenko and Clutterbuck, 1995, 1997; *Phanerochaete chrysosporium*, Rao and Reddy, 1984; and *Ashbya gossypii*, Schade et al., 2003) that may be used for increasing gene copy numbers to boost product yields. The recent introduction of *A. gossypii* as a potential production host for recombinant proteins (Ribeiro et al., 2010, 2013; Ribeiro, 2012) has brought these elements back to the limelight.

Filamentous fungi that dominate the scene as recombinant production hosts are the asexually reproducing *A. niger*, *A. oryzae*, and *T. reesei*. Consequently, most information on heterologous protein expression has come from studies using these particular fungi as well as the genetically well-characterized* A. nidulans*. A more recent contender for production of homologous and heterologous recombinant gene products is *Chrysosporium lucknowense* developed by Dyadic International Inc (Jupiter, FL, USA). Described advantages of the *C. lucknowense* system are high transformation frequencies, production of proteins at neutral pH, low viscosity of the fermentation broth due to specific strains and short fermentation times (Punt et al., 2001; Emalfarb et al., 2003). In this paper, we will concentrate on the work carried out with *T. reesei *that can be translated to other relevant filamentous fungi with relative ease.

Despite of the considerable amounts of work dedicated to the topic and rapid development of new techniques, there have been no big (published) break-throughs over the last few decades in terms of pushing the yields of heterologous gene products to the level of homologously produced proteins. So, what are we missing and where to look next?

## THE UNDERLYING EFFECTS OF RANDOM MUTAGENESIS

Filamentous fungi, have been developed as high-level enzyme producers for over thirty years, first using random mutagenesis and screening and more recently, genetic engineering (reviewed in Nevalainen et al., 2005; Ward, 2012). The history of strain improvement has been well documented for *T. reesei* (Durand et al., 1988; Peterson and Nevalainen, 2012). As classical genetic studies are not possible or not set up for the majority of the popular production hosts such as *A. niger* and *T. reesei*, even though the latter has a sexual stage (*Hypocrea jecorina*) and the former has a close relative *A. nidulans* for which the genetics is well known, the exact nature of the genetic constitution of hyperproducing strains remained unknown until genome sequencing became available. For example, sequencing of the genomes of a series of high cellulase-producing mutant strains of *T. reesei* revealed considerable changes in their genetic makeup compared to the wild-type QM6a (Martinez et al., 2008; Seidl et al., 2008; Le Crom et al., 2009; Vitikainen et al., 2010; reviewed in Peterson and Nevalainen, 2012; and Kubicek, 2013) and explained some of the observed phenotypic and metabolic characteristics; even so, the basis for drastically improved protein secretion remains unresolved.

Some of the high-protein producing strains such as *T. reesei* RutC-30 (Montenecourt and Eveleigh, 1979) are routinely used as expression hosts for homologous and heterologous gene products (reviewed in Mäntylä et al., 1998; Peterson and Nevalainen, 2012). One proposed foundation for efficient protein (cellulase) synthesis and secretion in this strain is an increased content of endoplasmic reticulum (ER), which provides more volumetric space for the synthesis of secreted proteins (Ghosh et al., 1982). This finding ultimately lead to the concept of “freeing up” space in the secretory pathway by deleting the genes encoding the major secreted proteins, Cellobiohydrolase I (CBHI/Cel7A), Cellobiohydrolase II (CBHII/Cel6A), and Endoglucanase I and II (EGI/Cel7B and EGII/Cel5A) in the case of *T. reesei *resulting in strains missing these genes in different combinations (Seiboth et al., 1992; Karhunen et al., 1993; Suominen et al., 1993; Wang et al., 2004; Rahman et al., 2009). In theory, eliminating the CBHI protein from the secretory pathway should free up about 60% of the capacity (Nummi et al., 1983; Harkki et al., 1991). While the operation has been moderately successful in some cases, in some others deletion of the gene encoding a major secreted protein has made no difference to the yield of a secreted recombinant protein (Miettinen-Oinonen et al., 1997). Deletion of the *cbh1* gene has been executed by targeted replacement of the endogenous *cbh1* locus with the gene of interest (e.g., Karhunen et al., 1993; Joutsjoki, 1994; Saarelainen et al., 1997; Miettinen-Oinonen and Suominen, 2002) with the presumed advantage of expressing the desired gene from a locus with high transcription efficiency. As an example, targeted replacement of the *cbh1 *locus of the high cellulolytic strain VTT-D-79125 with the endogenous *egl1*, followed by targeted replacement of the *cbh2 *locus with *egl2* resulted in a four-fold increase in EGI activity (Miettinen-Oinonen and Suominen, 2002). There is a lot less published information available for *Aspergillus*, perhaps because of high commercial sensitivity.

It seems evident that identifying individual genes and changes in the genomes will not provide an answer to the pending question of secretion supremacy. More likely, the answer will hide in complex interactions between relevant genes and proteins and their regulation. Also, maybe high cellulase-secreting *T. reesei* strains produced by random mutagenesis and screening have been already “conditioned” for cellulase production and secretion so that introducing a protein of a different nature to be made and secreted in high yields may not be as straightforward as it looks. Considering the above, it might be worthwhile to start again by introducing the gene of interest first and applying random mutagenesis and screening afterward to boost the production levels of the desired protein by mutations “matching” the requirements of this particular protein. Automated high-throughput screening programs will make screening of hundreds of thousands of mutants feasible on a case-by-case basis. The principle of “transformation first, screening second,” has been introduced using *Ashbya*. Random mutagenesis by ethyl methane sulfonate was carried out on *A. gossypii *transformants harboring the *T. reesei egl1 *gene, resulting in a global increase in protein secretion and a twofold to threefold increase in extracellular EGI activity (Ribeiro et al., 2013). Banking on random mutagenesis involving the entire genome to achieve a concerted effect to enhance synthesis and secretion of a gene product of interest, made in a transformant host seems attractive. For example, we do not have (as yet) information of all the genes involved in the process of efficient secretion of a gene product and even if we did, the current targeted approach through gene transformation and inactivation together with the requirement for marker recycling would propose a huge effort to deal with hundreds or so of genes probably involved with the process. Doing this by applying the methods of synthetic biology that allows re-engineering of entire pathways seems possible in the not so far future.

## ALTERNATIVE PROMOTERS AND TRANSCRIPTION FACTORS

The strong wild-type *cbh1* promoter encoding the major cellulase (CBH1/Cel7A) in *T. reesei* is the “default” promoter for recombinant gene expression (e.g., Harkki et al., 1991; Nyyssönen et al., 1993; Nykänen et al., 2002; Haakana et al., 2004; Nevalainen et al., 2005). While good yields have been obtained using this promoter, it has also turned out that the expression levels, especially those of heterologous proteins, may cause conformational stress to the production organism (Collén et al., 2005; Godlewski et al., 2009; Nykänen et al., in preparation). With a view of feeding a recombinant protein through the secretion pathway in a more uniform manner, some other, mainly constitutive promoters functional on glucose have been explored.

Isolation of *T. reesei* promoters that function on glucose has been described by Nakari et al. (1993; e.g., *tef1* encoding transcription elongation factor 1, and *hfb1 *encoding hydrophobin) and Curach et al. (2004; * hex1*); however, there seems to be no published information on the use of these promoters for the expression of recombinant proteins. The *tef1* and *hfb1 *promoters were isolated by a cDNA approach while the* hex1* promoter sequence was captured by chromosome walking, based on amino acid sequences from the HEX1 protein identified as one of the major proteins on a secretome of *T. reesei* grown on glucose (Lim et al., 2001). These different approaches introduce proteomic analysis as a tool for discovering and identifying promoters of highly expressed genes though identification of abundant proteins produced under defined conditions. In a recent study, Li et al. (2012) carried out transcriptional RT-qPCR profiling of 13 genes that were part of glucose metabolism in *T. reesei* QM9414 (Mandels et al., 1971). The promoters of *pdc* (pyruvate decarboxylase), *eno *(enolase), *gpd* (glyceraldehyde-3-phosphate dehydrogenase), *tpi* (triose phosphate isomerase), *pda* (pyruvate dehydrogenase), and *kdh* (ketoglutarate dehydrogenase) genes were singled out and proposed as candidates for constitutive expression of recombinant proteins. The *pdc *and *eno *promoters were further used for recombinant expression of the homologous *T. reesei xyn2* gene resulting in the production of 1.61 and 1.52 g/L of xylanase 2 respectively on glucose-containing medium (Li et al., 2012). The result can be considered promising as about similar amounts of xylanase 2 is expressed under its own promoter but on a cellulose-containing medium used for induction of the *xyn2* promoter.

There is continuing interest in studies into transcription factors participating in gene expression (Kiiskinen et al., 2004; Coradetti et al., 2012). While this line of research may help modulating regulation of gene expression and finding suitable and flexible cultivation conditions tailored for the gene promoter in play, it may not solve the problem of loss of the gene product during secretion.

On the note of transcription factors, it has been shown that the presence of multiple copies of one promoter can lead to the depletion of specific transcription factors for that promoter (Verdoes et al., 1994; Margolles-Clark et al., 1996). This situation may be avoided by expression of the gene of interest simultaneously under multiple different promoters inducible under the same conditions but only partly sharing the regulatory factors (Te’o and Nevalainen, 2008; Miyauchi et al., 2013).

## TRACKING PROTEIN SECRETION

**FIGURE 1:**
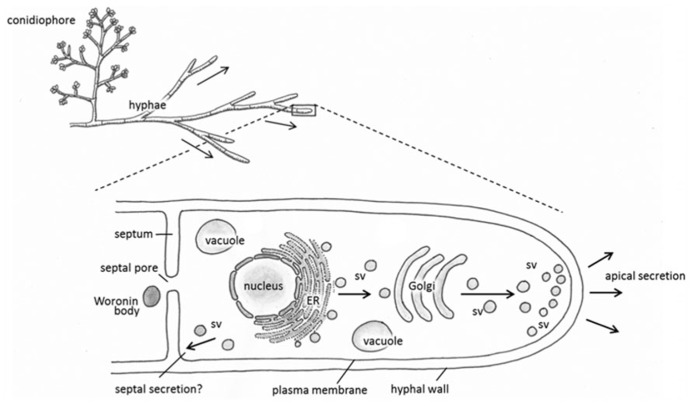
**Schematic representations depicting the general morphology of *Trichoderma reesei* (top) and proposed pathways of protein synthesis and secretion (enlarged hyphal tip, below).** Proteins are synthesized in the endoplasmic reticulum (ER), then travel in secretory vesicles (sv) to the Golgi for further post-translational modification. Secretory vesicles then carry the modified proteins to the hyphal tip for apical secretion, or possibly to the septa in an alternative secretory pathway.

It has been well established that the majority of secreted proteins including heterologous gene products are secreted through the growing hyphal tip (Wessels, 1993; Kiep et al., 2008; **Figure 1**). This default pathway is effective, for example, in the secretion of the glucoamylase enzyme in *A. niger* (Wösten et al., 1991) and cellobiohydrolase I in *T. reesei*. The ability of *T. reesei *hyphae to synthesize and secrete tens of grams and more of CBHI per liter of the cultivation medium could perhaps be explained further by assuming that there are supplementary mechanisms operating in the hyphae in addition to secretion via hyphal apices. Indeed, it has been shown that the CBHI enzyme is secreted also from the more mature parts of hyphae (Nykänen, 2002). In support of this view, *T. reesei* EGI and some heterologous enzymes such as *Hormoconis resinae* glucoamylase P and calf chymosin also occurred from mature parts of the *T. reesei* hyphae (Sprey, 1988; Nykänen, 2002). Contrary to these observations, secretion of the heterologous barley cysteine proteinase EPB seemed to occur solely at the hyphal tip thereby following the default pathway in *T. reesei* (Nykänen et al., 1997). These studies imply that there are spatial restrictions in secretion of foreign proteins that may be protein-dependent. Some factors contributing to this include subcellular localisation of the specific mRNA, information printed in the amino acid sequence of a protein and protein glycosylation.

A recent study with EGFP-fused alpha amylase in *A. oryzae* showed constitutive exocytosis also takes place at septa in addition to hyphal tips (Hayakawa et al., 2011). The fusion protein accumulated was shown to rapidly accumulate in the septal periplasm in a process that involved fusion of the secretory vesicles with the septal plasma membrane. Unlike exocytosis through hyphal tips, the process required microtubules but not F-actin whereas secretion through the tips requires both. Exocytosis toward septa may thus provide an interesting alternative to improve the production of secretory enzymes using filamentous fungi considering that in the industrially exploited species of *Aspergillus *and *Trichoderma*, there are far more septa than hyphal tips.

Recent confocal microscopy and ultrastructural studies into protein secreting *T. reesei* hyphae demonstrate a progressively changing spatial organization of the ER in response to secretion stress (Nykänen et al., in preparation). It has also provided ultrastructural evidence to support information obtained from genome sequencing. For example, the highly cellulolytic *T. reesei* RutC-30 mutant has identified deletions or mutation in genes encoding proteins associated with vesicle trafficking, vacuolar sorting and Golgi associated vacuolar ATPases (Le Crom et al., 2009) which play a role in protein secretion. These types of observations should be taken into account when choosing an expression host with an assessment of the level and type of secretion stress the strain is under before expression of a recombinant protein (Kautto et al., 2013).

To add to the importance of exploring the physiology of the expression host and protein yields comes from the work with *A. oryzae* where disruption of the vacuolar protein sorting receptor gene (Aovps10) enhanced production and secretion of both bovine chymosin and human lysozyme by 3- and 2.2-fold respectively (Yoon et al., 2010).

A recently described phenomenon contributing to protein externalization from the fungal hyphae is the “pulsing” mode of secretion noted for the highly expressed CBHI in the high cellulase-producing mutant strain *T. reesei* Rut-C30 (Godlewski et al., 2009). The pulsing may reflect physiological adjustment of the hyphae to the protein overload through membrane recycling and reorganization of the ER subdomains (Godlewski et al., 2009). This view now has support from studies by Nykänen et al. (in preparation) who have described the ER as a highly dynamic organelle of which the subdomains undergo structural changes according to the protein load in the ER. The pulsing was less evident when the heterologous bacterial enzyme Xylanase B (XynB) was expressed in *T. reesei*; instead, the heterologous protein seemed to continuously accumulate in the hyphae (Godlewski et al., 2009).

The sporadic nature of the studies into visualization and localisation of protein accumulation in the fungal hyphae makes drawing broader conclusions hard. However, there are clear indications that, once again, homologous and heterologous proteins are treated differently. According to current practice, the majority of heterologous recombinant proteins are produced as a fusion to an endogenous highly secreted protein such as the main cellobiohydrolase CBHI in *T. reesei*, assumed to function as an aid in the synthesis and secretion of a recombinant protein. One of the less noted, proposed functions of the endogenous fusion protein may be stopping the non-native protein from sticking to the cell wall (Nykänen, 2002).

## CONTAINED PROTEIN PRODUCTION

Proteins traveling through the fungal secretory pathway are relatively exposed to their environment. There have been some attempts toward “contained” production of secreted proteins in fungi. Naturally occurring plant protein bodies, derived from vacuoles or ER, represent a stable form of protein accumulation for nutrient storage in seeds. The ability of the maize storage protein Zera to induce the formation of protein bodies has been utilized for recombinant protein production in *T. reesei* (Torrent et al., 2009). A green fluorescent protein (GFP)-Zera peptide fusion accumulated in induced protein body-like organelles in the hyphae, protecting the recombinant fusion protein from cellular degradation whilst also protecting host cell viability. In addition, downstream isolation of the fusion protein was enhanced by the high density of the Zera-induced protein bodies.

Expression of a GFP fusion with the homologous hydrophobin I (HFBI) of *T. reesei *also induced the formation of protein bodies when targeted to the ER using the HDEL ER-retention signal (Mustalahti et al., 2013). A dual benefit was achieved; large ER-derived protein bodies containing soluble fusion protein accumulated in the hyphae, and the hydrophobicity of HFBI enabled effective downstream purification via a simple aqueous two-phase liquid partitioning system (ATPS). Purification by ATPS can be carried out relatively cheaply and simply even in large scale systems, as demonstrated by the successful purification of EGIcore-HFBI fusion protein from a 1200 l fermenter culture of a recombinant *T. reesei *strain (Collén et al., 2002; Selber et al., 2004).

## DECORATING PROTEINS WITH SUGARS

Glycosylation is one of the most common post-translational modifications and nearly 50% of all known proteins in eukaryotes are glycosylated (Apweiler et al., 1999). Glycans are synthesized by the coordinated action of glycosyltransferases, glycosidases, and other glycan processing enzymes. *N*-linked glycans have a role in many physiological and pathological events including protein and cell trafficking, immunogenicity, cell growth and adhesion, differentiation, tumor invasion, transmembrane signaling and host-pathogen interactions (Zhao et al., 2008).

Studies into the effect of glycosylation on the secretion, stability and activity and binding of secreted proteins have been carried out mainly with non-recombinant *T. reesei* cellulases. The various approaches and outcomes have been summarized in a recent review by Beckham et al. (2012). In the main, it has turned out that glycosylation has shown to have an effect on enzyme stability (aggregation and thermal stability) and activity. Contribution of glycosylation on protein secretion in filamentous fungi is less well established as some secreted native and recombinant proteins seem not to carry any glycan structures (Ülker and Sprey, 1990; Kurzatkowski et al., 1996; Paloheimo et al., 2003).

It has also been established that different fungi and fungal strains *N*-glycosylate proteins differently (Nevalainen et al., 1998) and that composition of the cultivation medium affects the glycosylation pattern (Stals et al., 2004). Despite of these leads, there are only a handful of papers comparing production of heterologous proteins in different host strains of the same fungal species (e.g., Bergquist et al., 2004) and different growth conditions, not to mention detailed analysis of the glycan structures attached on recombinant proteins. One such paper is that of Miyauchi et al. (2013) where the authors showed that the recombinant Xylanase B protein (from a thermophilic bacterium), produced in *T. reesei* featured multiple forms of the enzyme, decorated with various *N*- and *O*-glycans as assessed by mass spectrometry. One of the *O*-glycans was identified as hexuronic acid, which has not been described previously in the glycosylation patterns of *T. reesei.*

On the note that glycans have an effect on the activity of a protein, one of the problems standing in the way for filamentous fungi becoming effective producers of pharmaceutical proteins targeted for human consumption is the fungal oligo-mannose type glycosylation. While still of high-mannose type *N*-glycosylation patterns, filamentous fungi are far more conservative than yeast that has the tendency to hyperglycosylate proteins (Deshpande et al., 2008). However, they still lack the terminal sialic acid residues, characteristic of human glycosylation and important for defining the function of the glycan. These shortcomings have been addressed in a handful of *in vivo* studies toward modification of the protein glycosylation pathway in filamentous fungi, mainly *A. nidulans*, *A. niger*, *A. oryzae* (Kasajima et al., 2006; Kainz et al., 2008) and *T. reesei* (Maras et al., 1999; Zhong et al., 2011). Compared to yeast, activity in this field is very low.

Alternatively to humanizing the fungal glycosylation pathway, filamentous fungi can be considered a potential option for producing selected glycan-modifying enzymes for *in vitro* modification of glycans attached to recombinant proteins. The “strip and tease” approach is currently being developed in our laboratory (unpublished work). While these scenarios are not fully developed yet, the exceptional protein secretion capacity of filamentous fungi warrants investigation into modification of protein glycosylation in order to make functional therapeutic proteins in these organisms in an economically sustainable manner. Work in yeast provides a guide for these efforts.

## CURRENT PRODUCT LEVELS OF RECOMBINANT PROTEINS AND CLOSE COMPETITORS

Recent developments in mammalian cell culture have raised the production levels of heterologous pharmaceutically important proteins to grams per liter (Aldridge, 2006) reaching the yield of 26 g/L of a monoclonal antibody in an industrial setting (Jarvis, 2008). In comparison, published yields for antibodies produced in filamentous fungi are of the order of 0.15 g/L of a CBHI-Fab fusion antibody for *T. reesei* (Nyyssönen et al., 1993) and 0.9 g/L of Trastaztmab for *A. niger* (Ward et al., 2004). While filamentous fungi may have lost this battle, they still hold a good position as producers of various industrial enzymes as the mammalian systems are far too expensive and impractical for the bulk production of low-cost recombinant proteins.

Another near competitor is the methylotrophic yeast *Pichia pastoris* with production levels of 1.6 g/L for a monoclonal antibody in a glycoengineered strain (Ye et al., 2011). As *Pichia* is also capable of efficient secretion and the system is commercially available^1^, it offers a competitive edge when choosing a host for the production of heterologous proteins. Overall, it seems ever so important that the matching of the intended product and the production host is done with care and using all available information as the yields for different types of recombinant proteins can vary considerably even within the same host organism (see the review of Demain and Vaishnav, 2009).

## THE EFFECT OF FERMENTATION CONDITIONS

A typical workflow aiming at improvement of the product yield has a front-end involving optimization of the gene encoding the product of interest, the expression vector and the transformation method, and adding or removing tags for protein targeting and purification. Nature of these adjustments depends on the chosen production host. The next step is screening of the transformant strains for the desired product and making the recombinant product on a laboratory scale. This involves establishment of the cultivation parameters. It should be noted that different recombinant strains may require slightly different cultivation conditions and a protocol that differs from the transformation host (Sun, 2013). This aspect is often not studied in detail especially at the stage when a high number of genetically modified strains is screened. Therefore, some potentially good producers maybe lost in the “standardized” screening process.

Developing the cultivation conditions including the growth medium is fundamental for the improvement of the yields of recombinant proteins. This has been well established and documented with both mammalian cell cultures (e.g., Aldridge, 2006) and the *Pichia* system (e.g., Gonçalves et al., 2013) where the improvements have been impressive. There is also a wealth of published work on the development of protocols and models for growing fungi in submerged cultures, addressing the nature of the carbon and nitrogen sources, carbon:nitrogen ratio, agitation, aeration, nutrient depletion and feeding, to mention some (reviewed in Workman et al., 2013). Cultivation by solid fermentation and mass screening of fungal strains have also been described in the literature (reviewed e.g., in El-Enshasy, 2006).

Optimization of the production conditions for industrial scale fermentations are typically carried out in-house and patented.

## METABOLIC ENGINEERING AND SYNTHETIC BIOLOGY

Metabolic engineering and the application of synthetic biology are heavily reliant on the breadth and depth of large-scale (“omics”) information available for the organism of interest. This information comes from genome sequences, studies into metabolic pathways and fluxes, transcriptomic and proteomic data and bioinformatic modeling. Amongst these, quantification of metabolic fluxes is of utmost importance to understand biological networks for cellular regulation, and identify bottlenecks in product formation. The potential targets in filamentous fungi may include production of hydrolytic enzymes, organic acids, biofuels and chemicals. For example, the information gathered from the analysis of metabolic fluxes under production conditions will point out locations where the flow of, e.g., carbon in the cell is not going effectively toward the intended product thus proposing a bottleneck. This situation may then be remedied by genetic engineering by redirecting or enhancing the flow resulting in an improved product yield.

Genome sequences are available for a good amount of filamentous fungi including the industrially relevant *A. niger*^2^ and *A. oryzae*^3^, *P. chrysosporium*^4^ and *T. reesei *(wild-type and mutant strains.^5^. Application of targeted metabolic engineering to improve recombinant protein production has been carried out in bacterial and yeast systems and *Aspergillus* (reviewed by Melzer et al., 2009; Boghigian et al., 2010; Matsuoka and Shimizu, 2010). However, although metabolic engineering has been successfully employed to improve homologous cellulase production in *T. reesei* (Kubicek et al., 2009), little published information is available on its application to recombinant *T. reesei *strains to date.

A high throughput gene deletion in *T. reesei *has recently been developed (Schuster et al., 2012). A series of selection markers were used to provide a primer database for gene deletion, and vector construction was carried out by yeast- mediated recombination. Transformation of the vector into a *T. reesei *strain deficient in non-homologous end joining (NHEJ) was followed by crossing of mutants with sexually competent strains to remove the NHEJ-defect.

Synthetic biology takes matters further with the goal of designing and constructing biological devices and systems. This would involve rearranging and rebuilding large DNA constructs with overlapping DNA fragments and their *in vivo* recombination (Gibson et al., 2009). The strategy has been shown to work well with bacteria and yeast but there are no published reports concerning filamentous fungi as yet. One of the future scenarios may feature mini-cell factories where only the essential functions for making a particular gene product are contained.

## CONCLUSION

Filamentous fungi offer enormous potential for efficient and large scale production of recombinant gene products. Importantly, protein secretion provides a platform for the eukaryotic style post-translational modification of proteins. Fungi are cheap to cultivate and down-stream processing is made easy with no need to break cells open for product recovery. In order to capitalize on fungi as recombinant production hosts, research is now directed to revealing the cellular mechanisms for internal protein quality control, secretion stress, functional genomics of protein expression and secretion, protein modification and linking the physiology to productivity. New directions are expected to emerge from overlaying the “omics” data and the rapidly developing technologies of metabolic engineering and synthetic biology. Our long held expectations of filamentous fungi as high-level producers of a wide range of recombinant proteins may well be drawing closer to full realization. Or, perhaps, the future may see a change in the expectations and a focusing toward specific areas of recombinant expression for which the fungal system is particularly adept, such as the production of recombinant enzymes including therapeutic microbial enzymes.

## Conflict of Interest Statement

The authors declare that the research was conducted in the absence of any commercial or financial relationships that could be construed as a potential conflict of interest.

## Footnotes

1 http://www.lifetechnologies.com

2 http://genome.jgi-psf.org/Aspni.home.html

3 http://www.bio.nite.go.jp/dogan/project/view/AO

4 http://genome.jgi-psf.org/whiterot/whiterot1.home.html

5 http://genome.jgi.doe.gov/genome-projects/pages/projects.jsf?searchText=Trichoderma+reesei
